# The Role of Oxidative Stress in the Effect of Quercetin on Na^+^/K^+^-ATPase Expression in Skeletal Muscle in a Metabolic Syndrome Model

**DOI:** 10.3390/ijms27104369

**Published:** 2026-05-14

**Authors:** Ayca Bilginoglu Topcu

**Affiliations:** Ankara Yildirim Beyazit University, Faculty of Medicine, Department of Biophysics, Ankara 06800, Türkiye; abilginoglu@aybu.edu.tr

**Keywords:** metabolic syndrome, NKA, quercetin, skeletal muscle, oxidative stress

## Abstract

Metabolic syndrome (MeS) is a multifactorial disorder characterized by insulin resistance, dyslipidemia, hypertension, and obesity, and oxidative stress plays a key role in tissue damage in this syndrome. This study aimed to investigate this role in Na^+^/K^+^-ATPase (NKA) expression in skeletal muscle and to evaluate the effects of quercetin. A high-sucrose-diet-induced MeS model was established in Wistar albino rats (*n* = 32), and skeletal muscle tissues were analyzed. Biochemical parameters were measured, including aspartate aminotransferase (AST), lactate dehydrogenase (LDH), total antioxidant status (TAS), total oxidant status (TOS), superoxide dismutase (SOD), and malondialdehyde (MDA). In addition, thioredoxin-1 (TRX1) and NKA protein expression levels were evaluated using Western blot analysis. In the MeS group, AST, TAS, TRX1, and NKA expression significantly decreased, while LDH, TOS, SOD, and MDA levels increased, indicating disrupted redox balance, elevated oxidative stress, and impaired antioxidant defense. Increased MDA and TOS levels reflected enhanced lipid peroxidation, whereas decreased TAS and TRX1 suggested reduced antioxidant capacity. Elevated SOD activity may indicate a compensatory response to excessive reactive oxygen species (ROS). The reduction in NKA expression may contribute to impaired ion transport and potential skeletal muscle dysfunction. Quercetin administration improved oxidative stress markers and partially restored NKA expression. These findings suggest that oxidative stress contributes to NKA dysfunction in MeS, and quercetin may have therapeutic potential by modulating oxidative stress and preserving enzyme function.

## 1. Introduction

MeS is a complex metabolic disorder characterized by insulin resistance, obesity, dyslipidemia, and hypertension, leading to functional and biochemical alterations in multiple tissues, particularly skeletal muscle [1]. Skeletal muscle plays a central role in both energy metabolism and glucose homeostasis, making it a critical target tissue in the pathogenesis of metabolic syndrome. In this context, NKA, a key enzyme responsible for maintaining ion balance and cellular excitability in muscle cells, is directly affected by metabolic disturbances [2].

Accumulating evidence indicates that oxidative stress is significantly increased in metabolic syndrome, resulting in damage to cellular proteins, lipids, and enzymatic systems. In particular, increased production of ROS leads to elevated TOS and decreased TAS, reflecting a disruption in cellular redox balance [3]. In parallel, increased levels of MDA, an important marker of lipid peroxidation, and decreased levels of TRX, a critical component of the antioxidant defense system, further indicate the progression of oxidative damage [4].

In metabolic syndrome, increased SOD activity is considered a compensatory response to elevated oxidative stress. However, despite this adaptive response, the detrimental effects of excessive oxidative burden on cellular enzymes persist, with decreased NKA expression being a particular result. Previous studies have demonstrated that oxidative stress can suppress NKA expression through post-translational modifications such as glutathionylation, thereby impairing muscle function [5].

From a biochemical perspective, decreased AST levels and increased LDH levels observed in metabolic syndrome are considered important indicators of tissue damage and impaired energy metabolism [6]. Elevated LDH levels, in particular, reflect increased anaerobic metabolism and heightened cellular stress.

Quercetin, a naturally occurring flavonoid, has attracted considerable attention due to its strong antioxidant and anti-inflammatory properties and has emerged as a potential agent for reducing oxidative stress associated with metabolic syndrome. It has been reported that quercetin exerts protective effects by scavenging free radicals, regulating antioxidant enzyme systems, and modulating cellular signaling pathways [7]. Furthermore, several studies have demonstrated that quercetin can preserve NKA activity and improve oxidative stress-related enzymatic alterations [8,9].

Based on these considerations, this study aimed to investigate the role of oxidative stress in NKA expression in skeletal muscle in a metabolic syndrome model and to evaluate the potential modulatory effects of quercetin on this process.

## 2. Results

### 2.1. Influence of Quercetin on Metabolic and Biochemical Parameters in Experimental Groups

At the end of the experimental period, the MeS group exhibited significant increases in body weight, waist circumference, blood glucose, triglyceride levels, insulin levels, and homeostasis model assessment of insulin resistance (HOMA-IR) index compared to the control (Con) group (*p* < 0.05). In addition, high-density lipoprotein cholesterol (HDL-C) levels were significantly decreased in the MeS group (*p* < 0.05). Quercetin administration (Q) alone did not significantly alter these parameters in comparison to the control group (*p* > 0.05), indicating no adverse metabolic effect. In the MeS-Q group, quercetin treatment partially ameliorated metabolic disturbances induced by the high-sucrose diet. Specifically, waist circumference, triglyceride levels, and HDL-C showed significant improvement compared to the MeS group (*p* < 0.05). Although reductions were also observed in body weight, blood glucose, insulin levels, and HOMA-IR, these changes were not statistically significant (*p* > 0.05) (Table 1).

### 2.2. Assessment of AST and LDH Parameters Under Quercetin Intervention in MeS

**Figure 1:**
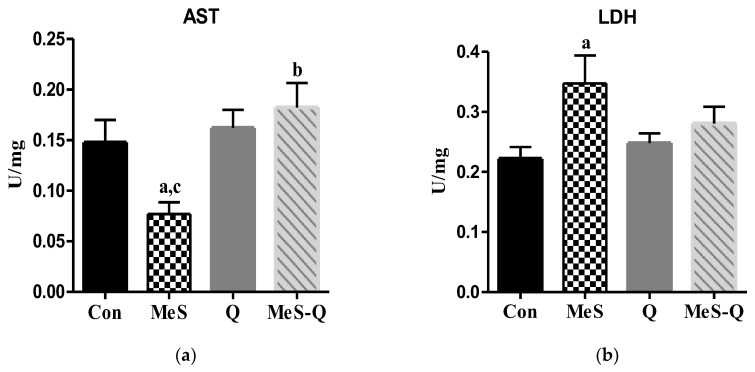
Oxidative metabolism enzyme activity in skeletal muscle tissues of experimental groups. (**a**) AST: Aspartate aminotransferase; (**b**) LDH: lactate dehydrogenase; Con: control group; MeS: metabolic syndrome group; Q: quercetin-treated group; MeS-Q: quercetin-treated metabolic syndrome group. Values are represented as mean ± standard deviation (SD). The number of rats in each group is eight. ^a^ *p* < 0.05 vs. Con; ^b^ *p* < 0.05 vs. MeS; ^c^ *p* < 0.05 vs. Q.

AST levels in skeletal muscle were markedly reduced in the MeS group relative to the Con group (*p* < 0.05), whereas no significant difference was observed between the Q and Con groups (*p* > 0.05). Notably, quercetin administration in the MeS-Q group significantly elevated AST levels (*p* < 0.01), bringing them closer to those of the Con group (Figure 1a). Similarly, LDH levels were significantly higher in the MeS group than in the Con group (*p* < 0.05). The Q group showed no significant change compared with the Con group (*p* > 0.05). Although LDH levels tended to decrease following quercetin treatment in the MeS-Q group, this reduction was not statistically significant (*p* > 0.05) (Figure 1b).

### 2.3. Alterations in Oxidative Stress and Antioxidant Status Following Quercetin Administration in MeS Conditions

**Figure 2:**
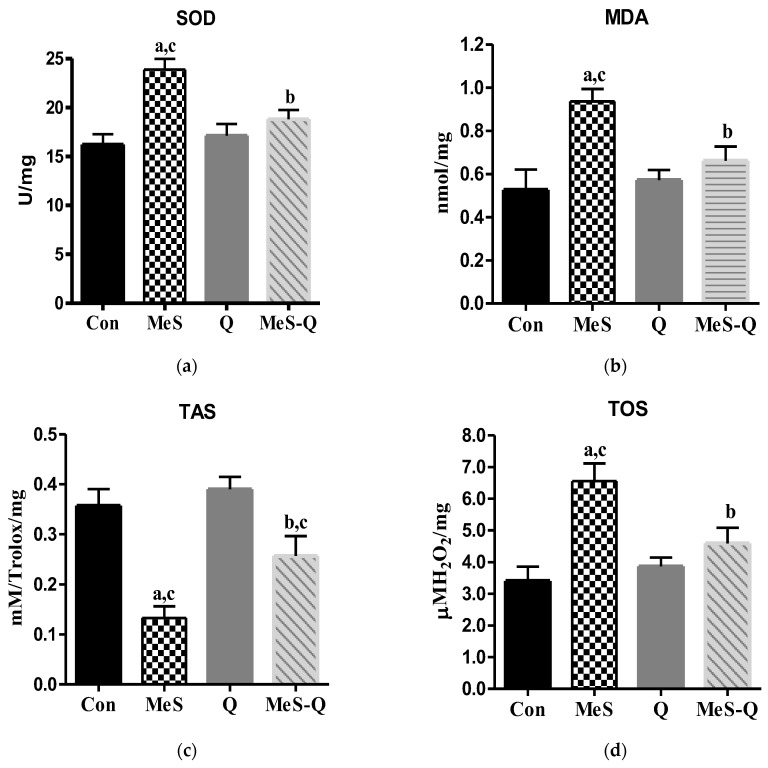
Oxidative stress and antioxidant status in skeletal muscle tissues of experimental groups. (**a**) SOD: Superoxide dismutase; (**b**) MDA: malondialdehyde; (**c**) TAS: total antioxidant status; (**d**) TOS: total oxidant status; Con: control group; MeS: metabolic syndrome group; Q: quercetin-treated group; MeS-Q: quercetin-treated metabolic syndrome group. Values are represented as mean ± standard deviation (SD). The number of rats in each group is eight. ^a^ *p* < 0.05 vs. Con; ^b^ *p* < 0.05 vs. MeS; ^c^ *p* < 0.05 vs. Q.

As shown in Figure 2a, SOD activity in skeletal muscle was markedly higher in the MeS group than in the Con group (*p* < 0.001). In contrast, the Q group showed values similar to or slightly below those of the Con group, while quercetin administration in the MeS-Q group significantly lowered SOD activity compared with findings in the MeS group (*p* < 0.01). Regarding MDA levels (Figure 2b), a significant increase was observed in the MeS group compared with Con (*p* < 0.01). The Q group showed reduced MDA levels relative to MeS, and quercetin treatment significantly decreased MDA levels in the MeS-Q group (*p* < 0.05). As shown in Figure 2c, TAS levels were significantly reduced in the MeS group compared with the Con group (*p* < 0.001), whereas the Q group exhibited higher TAS levels than the MeS group. Quercetin supplementation partially improved TAS levels in the MeS-Q group (*p* < 0.05 vs. MeS). Figure 2d illustrates that TOS levels were significantly elevated in the MeS group compared with Con (*p* < 0.001), while the Q group displayed lower TOS levels than MeS. Quercetin administration significantly decreased TOS levels in the MeS-Q group (*p* < 0.05).

### 2.4. Modulation of TRX1 Levels by Quercetin in MeS Rats

**Figure 3:**
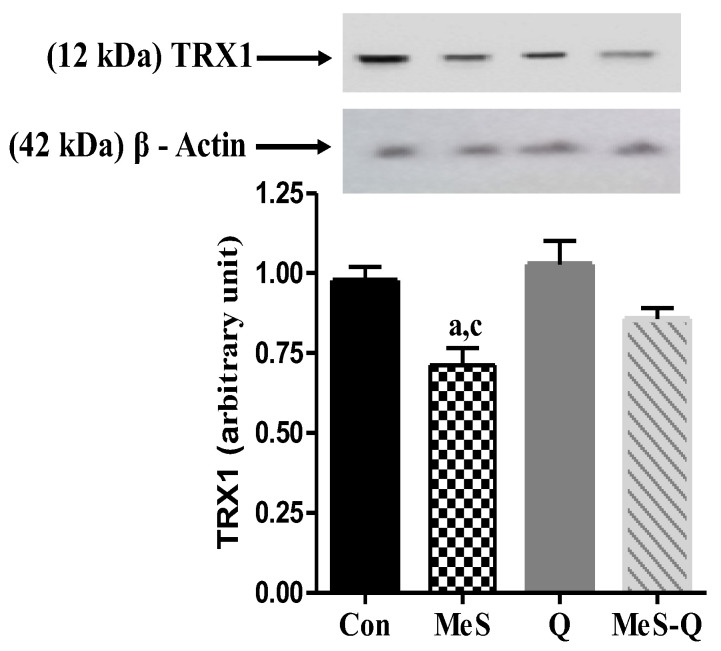
Thioredoxin1 (TRX1) levels in skeletal muscle tissues of experimental groups. Con: Control group; MeS: metabolic syndrome group; Q: quercetin-treated group; MeS-Q: quercetin-treated metabolic syndrome group. Values are represented as mean ± standard deviation (SD). The number of samples was three in each group (n = 3). ^a^ *p* < 0.05 vs. Con; ^c^ *p* < 0.05 vs. Q.

Figure 3 shows that TRX1 levels in the skeletal muscle of the MeS group were significantly decreased (*p* < 0.01) compared with the Con group. However, quercetin administration did not produce a statistically significant increase in TRX1 levels compared with the MeS group (*p* > 0.05), although a slight upward trend was observed. The Q group did not significantly alter this parameter compared to the Con group (*p* > 0.05).

### 2.5. Changes in NKA Expression Following Quercetin Administration in a MeS Model

**Figure 4:**
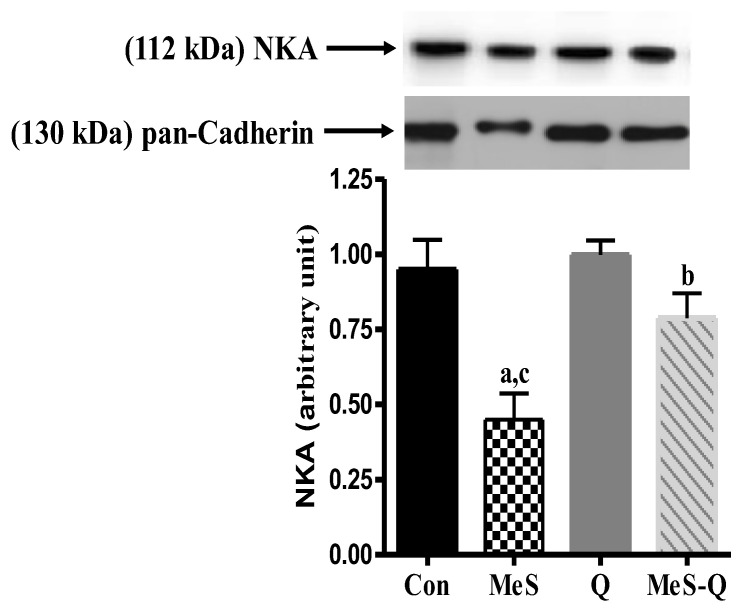
Na^+^/K^+^-ATPase (NKA) expression in skeletal muscle tissues of experimental groups. Con: Control group; MeS: metabolic syndrome group; Q: quercetin-treated group; MeS-Q: quercetin-treated metabolic syndrome group. Values are represented as mean ± standard deviation (SD). The number of samples was three in each group (n = 3). ^a^ *p* < 0.05 vs. Con; ^b^ *p* < 0.05 vs. MeS; ^c^ *p* < 0.05 vs. Q.

In Figure 4, NKA expression was significantly decreased in the MeS group (*p* < 0.01) compared with the Con group. However, quercetin administration significantly increased NKA expression (*p* < 0.05), bringing it closer to levels comparable to those of the control group. Notably, the Q group did not show a significant difference compared with the Con group (*p* > 0.05). Figure S1 shows the representative raw western blot images for NKA and TRX1.

## 3. Discussion

The present study was designed to investigate the role of oxidative stress in regulating NKA expression in skeletal muscle under metabolic syndrome conditions and to evaluate the modulatory effects of quercetin on this process. The findings provide clear evidence that metabolic syndrome-induced oxidative stress is closely associated with a reduction in NKA expression, indicating that redox imbalance is a critical determinant of membrane-associated enzyme function in skeletal muscle. Furthermore, the partial restoration of NKA expression following quercetin administration suggests that the modulation of oxidative stress can preserve enzyme integrity and contribute to the maintenance of cellular ion homeostasis.

The data obtained in this study support the concept that metabolic syndrome promotes a sustained shift toward a pro-oxidant intracellular environment. The observed increase in TOS and MDA levels, together with the reduction in TAS, reflects a disruption of redox balance characterized by enhanced oxidative burden and insufficient antioxidant defense capacity. This imbalance likely leads to cumulative oxidative damage to lipids, proteins, and membrane structures, ultimately impairing cellular function. Such alterations are particularly critical in skeletal muscle, given the tissue’s high metabolic activity and dependence on tightly regulated ion gradients [10].

The increase in SOD activity observed in the metabolic syndrome group appears to represent a compensatory response to elevated ROS production. However, despite this adaptive upregulation, the concomitant decrease in TAS and TRX1 levels suggests that endogenous antioxidant systems are insufficient to counteract persistent oxidative stress. This indicates that chronic metabolic stress exceeds the buffering capacity of intracellular defense mechanisms, leading to sustained redox imbalance and progressive cellular dysfunction [11,12].

A key mechanistic finding of the present study is the marked reduction in NKA expression under conditions of oxidative stress. NKA is highly sensitive to the redox status of the cellular environment, and its activity depends on both membrane integrity and the structural stability of its protein subunits [2]. Increased lipid peroxidation, as indicated by elevated MDA levels, may alter membrane fluidity and disrupt the optimal microenvironment required for NKA function. In parallel, the oxidative modification of protein thiol groups may induce conformational changes that impair enzyme activity [13]. These combined effects likely contribute to the observed downregulation of NKA and the disruption of ion homeostasis in skeletal muscle.

Beyond its classical function as an ion transporter, NKA also plays an important role in cellular signaling and metabolic regulation [8]. Therefore, its impairment may have broader consequences, including altered calcium handling, reduced mitochondrial efficiency, and impaired insulin signaling [14]. In this context, decreased NKA expression may represent a critical link between oxidative stress and metabolic dysfunction in skeletal muscle, contributing to reduced cellular performance and increased susceptibility to metabolic injury.

The alterations observed in AST and LDH levels further support the presence of metabolic and structural stress in skeletal muscle. Reduced AST levels may reflect impaired amino acid metabolism and mitochondrial dysfunction, whereas elevated LDH levels indicate a shift toward anaerobic glycolysis. This metabolic adaptation suggests reduced oxidative capacity and increased reliance on less efficient energy production pathways, which may further exacerbate cellular stress and functional impairment [6].

Another important observation of this study is the reduction in TRX1 levels, highlighting the involvement of thiol-dependent antioxidant systems in metabolic syndrome. The thioredoxin system is essential for maintaining protein redox balance and protecting against oxidative damage [15]. The limited response of TRX1 to quercetin treatment suggests that thiol-based antioxidant pathways may be less responsive to short-term intervention or may require more targeted or prolonged therapeutic strategies. This finding underscores the complexity of intracellular antioxidant networks and indicates that different components of the redox system may exhibit distinct sensitivities to oxidative stress.

Quercetin administration resulted in significant improvements in oxidative stress parameters, including reductions in TOS and MDA levels and partial restoration of TAS. These findings indicate that quercetin effectively modulates redox balance under metabolic stress conditions. The observed decrease in SOD activity following quercetin treatment may reflect a reduced need for compensatory antioxidant responses due to lower ROS levels [16,17,18,19]. This suggests that quercetin not only acts as a direct free radical scavenger but may also enhance overall cellular redox stability.

Importantly, the partial recovery of NKA expression in the quercetin-treated group indicates that reducing oxidative stress can help preserve membrane-associated enzyme function. However, the incomplete normalization of NKA suggests that its regulation is multifactorial. In addition to oxidative stress, factors such as ATP availability, membrane lipid composition, and hormonal regulation are likely to influence NKA expression and activity [20,21]. Therefore, antioxidant treatment alone may not be sufficient to fully restore enzyme function under metabolic syndrome conditions.

Overall, the findings of this study emphasize that increased oxidative stress plays a critical role in the reduction in NKA expression in metabolic syndrome and may contribute significantly to skeletal muscle dysfunction. The interplay between impaired antioxidant defenses, enhanced ROS production, and enzyme inactivation appears to be a key mechanism underlying these alterations. Quercetin emerges as a promising therapeutic agent capable of modulating oxidative stress and partially restoring cellular function in this context. Although individual roles of oxidative stress and quercetin have been previously reported, the present study provides an integrated evaluation of TRX1 expression, NKA expression, and tissue redox parameters in skeletal muscle within a high-sucrose diet-induced metabolic syndrome model, offering a more comprehensive view of membrane-associated redox regulation.

Nevertheless, further studies involving different doses and longer treatment durations are required to better elucidate the underlying mechanisms. In addition, well-designed clinical studies are necessary to translate these findings into human applications. Moreover, the absence of a vehicle-treated control group represents a limitation of the present study, which should be considered when interpreting the results. Furthermore, the lack of assessment of inflammatory markers is another limitation, and the inclusion of these parameters in future studies would provide a more comprehensive understanding of the underlying mechanisms.

## 4. Materials and Methods

### 4.1. MeS Rat Model

Three-month-old male Wistar albino rats (200–250 g) were used in this study and housed under controlled laboratory conditions, including a 12 h light/dark cycle, temperature of 24 ± 2 °C, and humidity of 35–60%. The animals were provided with standard laboratory chow and had unrestricted access to water.

At the end of the experimental period, all rats were fasted for 12 h and then anesthetized before sample collection. The animals were randomly assigned into three groups, each consisting of eight rats. The Con group received a standard diet and normal drinking water. The MeS group was given 32% sucrose (935 mM) in drinking water for 20 weeks to induce metabolic syndrome. The Q group received quercetin (15 mg/kg/day) via oral gavage for 20 weeks. The MeS-Q group received quercetin (15 mg/kg/day) via oral gavage for the final two weeks of the 20-week MeS induction period [22].

Body weight, blood glucose, insulin, triglyceride levels, HDL-C, HOMA-IR, and HOMA-β indices were measured in all groups as previously described [23]. All experimental procedures were approved by the Animal Ethics Committee of Ankara University Faculty of Medicine (2015-2-37; 11 February 2015).

### 4.2. Subcellular Fractionation from Skeletal Muscle Tissue

Plasma membrane and cytosolic fractions were isolated from skeletal muscle tissue using differential centrifugation. All procedures were performed at 0–4 °C. Fresh skeletal muscle tissue was rapidly excised, rinsed with ice-cold PBS to remove blood, and minced into small pieces on ice. The tissue was homogenized at a 1:10 (*w*/*v*) ratio in an ice-cold buffer containing the following (mM): Tris-HCl 20 (pH 7.4), NaCl 150, KCl 2, EDTA 2, DTT 0.5, PMSF 0.4, and 1% protease inhibitor cocktail (Sigma-Aldrich, St. Louis, MO, USA). Homogenization was performed using a Teflon-glass homogenizer. The homogenate was centrifuged at 1000× *g* for 10 min to remove nuclei and cellular debris (Optima™ XPN-100 Ultracentrifuge, Beckman Coulter, Brea, CA, USA). The supernatant (S1) was further centrifuged at 10,000× *g* for 15–20 min to eliminate mitochondria and heavy organelles. The resulting supernatant (S2) was subjected to ultracentrifugation at 100,000× *g* for 60 min, yielding a cytosolic fraction (S3) and a membrane-enriched pellet (P3). The cytosolic fraction (S3) was used for TRX1 protein analysis as well as biochemical measurements. The membrane fraction (P3) was resuspended and further purified by an additional washing centrifugation step at 100,000× *g* for 60 min and used for NKA protein analysis.

### 4.3. Evaluation of Oxidative Metabolism Enzyme Activity

Following the homogenization of skeletal muscle tissues, protein concentration was determined by the Bradford method (Bio-Rad Protein Assay, Bio-Rad Laboratories, Hercules, CA, USA), with bovine serum albumin used as the calibration standard. The activities of key metabolic enzymes, including AST and LDH, were measured in tissue samples using commercially available assay kits (AST Assay Kit and LDH Assay Kit; BioVision Inc., Milpitas, CA, USA, and Cusabio Technology LLC, Wuhan, China, respectively) according to the manufacturers’ instructions.

### 4.4. Assessment of Antioxidant Defense

SOD activity was determined using an enzymatic method based on the xanthine–xanthine oxidase system by monitoring the inhibition of nitroblue tetrazolium (NBT) reduction. SOD activity was defined as the amount of enzyme required to cause 50% inhibition of NBT reduction [24]. MDA levels were measured using the thiobarbituric acid (TBA) reactive substances (TBARS) method, and the absorbance of the resulting pink chromogen was measured spectrophotometrically at 532 nm (UV-1800 Spectrophotometer, Shimadzu, Kyoto, Japan) [25]. TOS and TAS were determined using commercially available assay kits (Rel Assay Diagnostics, Gaziantep, Türkiye) according to the manufacturer’s instructions.

### 4.5. Quantification of NKA and TRX1 in Skeletal Muscle Tissue Homogenates

NKA and TRX1 protein levels were analyzed by Western blotting in skeletal muscle subcellular fractions. NKA expression was assessed in the membrane-enriched fraction (P3), whereas TRX1 expression was determined in the cytosolic fraction (S3). For Western blot analysis, 20 μg of protein per sample was separated under reducing conditions using 10% SDS-PAGE. Following electrophoretic separation, proteins were transferred onto nitrocellulose membranes (Amersham Biosciences, Chicago, IL, USA) at 150 V for 1.5 h. Membranes were blocked with a 5% non-fat dry milk prepared in Tris-buffered saline containing 0.05% Tween-20. NKA and TRX1 expression were detected using rabbit polyclonal anti-NKA (1:5000, Abcam, Cambridge, UK) and anti-TRX1 (1:1000, Abcam, Cambridge, UK) antibodies, respectively. Immunoreactive bands were visualized using an enhanced chemiluminescence (ECL) detection system (Amersham ECL kit, GE Healthcare, Chicago, IL, USA).

### 4.6. Statistical Analysis

Statistical analyses were performed using GraphPad Prism (GraphPad Software, San Diego, CA, USA, version 8.0). Multiple comparisons were assessed using one-way analysis of variance (ANOVA) followed by Bonferroni post hoc tests. Data are presented as mean ± standard deviation (SD). Normality and homogeneity of variances were assessed using the Shapiro–Wilk and Levene’s tests, respectively, and all datasets met these assumptions. A *p*-value of less than 0.05 was considered statistically significant in all analyses.

## 5. Conclusions

In conclusion, this study demonstrates that metabolic syndrome induces significant oxidative stress in skeletal muscle, as evidenced by increased TOS, MDA, SOD, and LDH levels, along with decreased TAS, TRX, AST, and NKA expression. These alterations indicate a profound disruption in redox homeostasis and cellular metabolism. The decrease in NKA expression appears to be closely associated with oxidative stress-induced modifications, suggesting that impaired ion transport may contribute to skeletal muscle dysfunction in metabolic syndrome. The imbalance between oxidant production and antioxidant defense mechanisms plays a key role in this process. Quercetin, due to its antioxidant and regulatory properties, may help attenuate oxidative damage and restore enzyme function. Further experimental and clinical studies are required to better elucidate the underlying mechanisms and to determine the therapeutic potential of quercetin in metabolic syndrome-related skeletal muscle dysfunction.

## Figures and Tables

**Table 1:** Biochemical parameters in experimental groups.

Parameters/Groups	Con	MeS	Q	MeS-Q
Body Weight (g)	331.1 ± 15.7	442.1 ± 18.9 **^a^**	340.5 ± 15.2 ^b^	427.5 ± 20.2 ^a,c^
Waist circumference (cm)	10.7 ± 0.5	18.7 ± 0.4 ^a^	11.6 ± 0.7 ^b^	16.5 ± 0.8 ^a,c^
Blood glucose (mg/dL)	307.6 ± 8.4	429.6 ± 13.8 ^a^	323.3 ± 11.1 ^b^	410.1 ± 12.4 ^a,c^
Triglyceride (mg/dL)	29.4 ± 2.3	63.1 ± 3.0 ^a^	32.7 ± 2.1 ^b^	45.3 ± 2.7 ^a,b,c^
HDL-C (mg/dL)	55.7 ± 2.6	32.7 ± 1.7 ^a^	58.4 ± 2.5 ^b^	45.8 ± 2.6 ^a,b,c^
Insulin (ng/mL)	1.2 ± 0.1	2.6 ± 0.2 ^a^	1.1 ± 0.1 ^b^	2.3 ± 0.2 ^a,c^
HOMA-IR	12.2 ± 0.9	19.6 ± 2.1 ^a^	12.6 ± 1.2 ^b^	17.3 ± 1.1
HOMA-β	0.53 ± 0.02	0.48 ± 0.01 ^a^	0.50 ± 0.02	0.49 ± 0.02

HDL-C: High-density lipoprotein cholesterol; HOMA-IR: homeostasis model assessment of insulin resistance; Con: control group; MeS: metabolic syndrome group; Q: quercetin-treated metabolic syndrome group; MeS-Q: quercetin-treated metabolic syndrome group. Values are represented as mean ± standard deviation (SD). The number of rats in each group is eight. ^a^ *p* < 0.05 vs. Con; ^b^ *p* < 0.05 vs. MeS; ^c^ *p* < 0.05 vs. Q.

## Data Availability

The original contributions presented in this study are included in the article/Supplementary Materials. Further inquiries can be directed to the corresponding author.

## Supplementary Materials

The following supporting information can be downloaded at https://www.mdpi.com/article/10.3390/ijms27104369/s1.
