# Supplementary material for: The Role of Oxidative Stress in the Effect of Quercetin on Na+/K+-ATPase Expression in Skeletal Muscle in a Metabolic Syndrome Model
Source: Int J Mol Sci. 2026 May 14;27(10):4369. doi: 10.3390/ijms27104369 (PMC13207381; doi:10.3390/ijms27104369)
Supplement: Supplementary file 1 [file ijms-27-04369-s001.zip › ijms-4298334-supplementary.pdf]

The Role of Oxidative Stress in the Effect of Quercetin on Na^+/K^+-ATPase Expression in Skeletal Muscle in a Metabolic Syndrome Model

Ayca Bilginoglu Topcu

Department of Biophysics, Faculty of Medicine, Ankara Yildirim Beyazit University, Ankara 06760, Türkiye; abilginoglu@aybu.edu.tr or draycabilginoglu@hotmail.com

Tel.: +90 312 3241555/2065

Fax: +90 312 9062980

A)

Sample 1	Sample 2	Sample 3

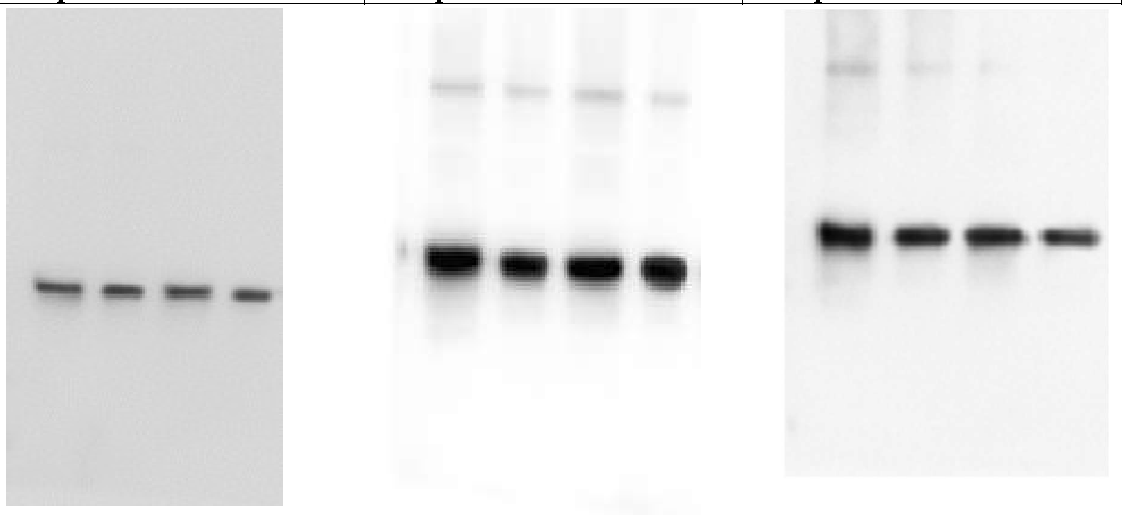

B)

Sample 1	Sample 2	Sample 3

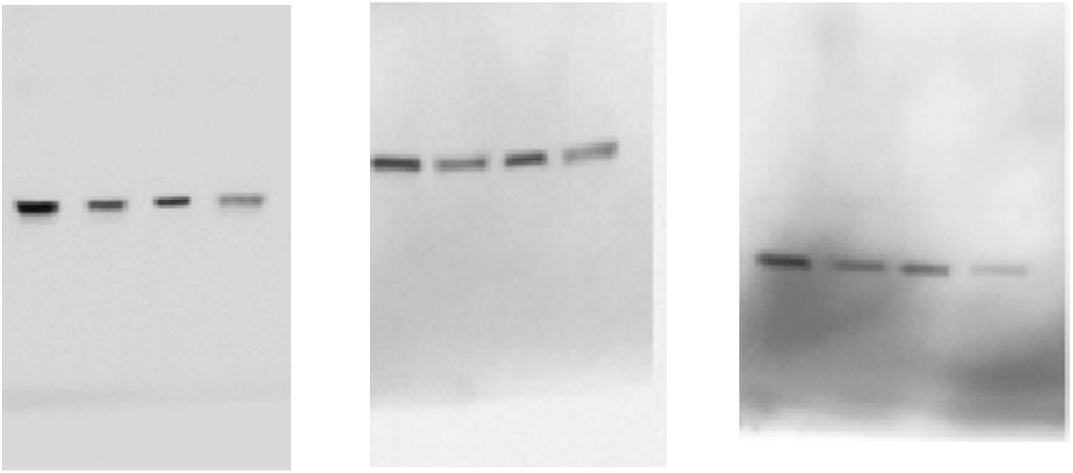

C)

Sample 1	Sample 2	Sample 3

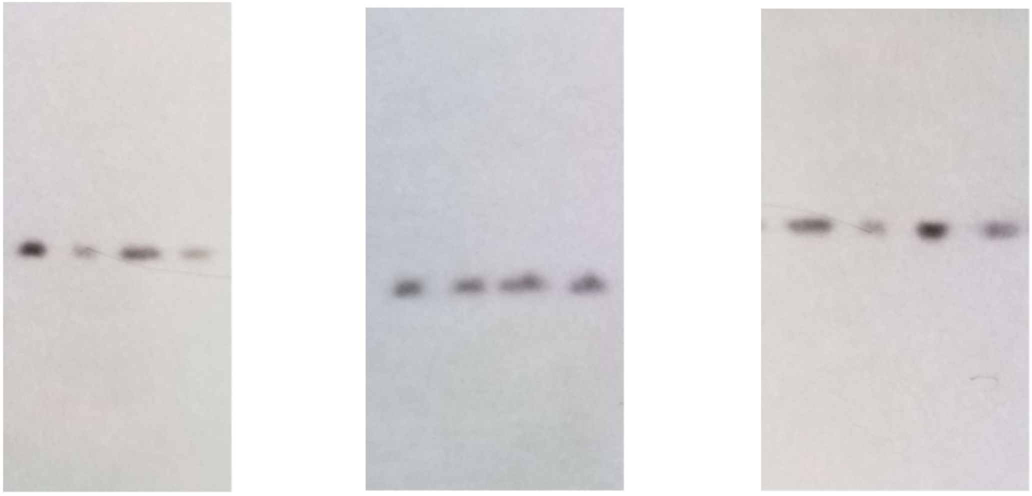

D)

Sample 1	Sample 2	Sample 3

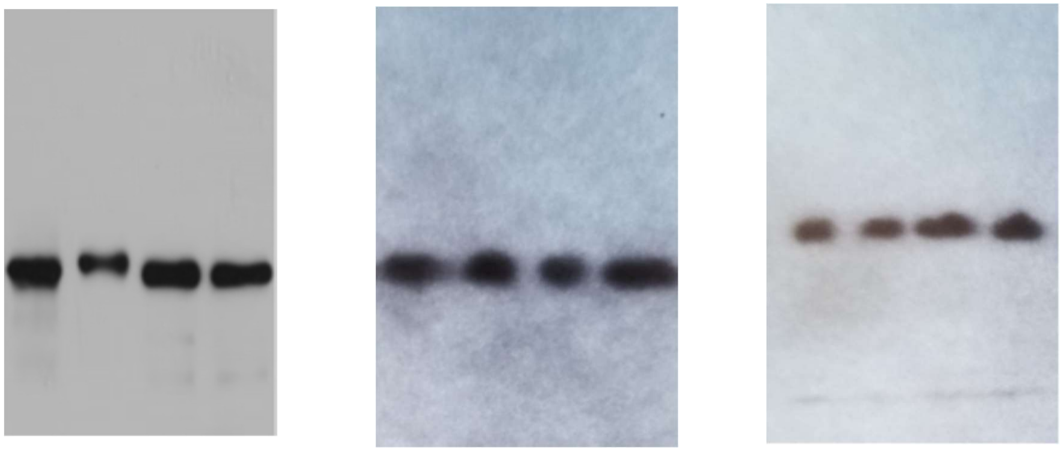

Figure S1. The row protein band expression data of NKA (**A**), TRX1 (**B**), β-Actin (**C**) and pan-Cadherin (**D**) in the four groups (Con, MeS, Q and MeS-Q).
